# Cerebral hemodynamic alterations in patients with Covid-19

**DOI:** 10.3906/sag-2006-203

**Published:** 2021-04-30

**Authors:** Ali Rıza SONKAYA, Bilgin ÖZTÜRK, Ömer KARADAŞ

**Affiliations:** 1 Department of Neurology, University of Health Science, Gülhane School of Medicine, Ankara Turkey

**Keywords:** Covid-19, SARS-Cov-2, blood flow velocity, vasomotor reactivity

## Abstract

**Background/aim:**

Coronavirus 2019 disease (Covid-19) was first seen in December 2019 and afterwards it became pandemic. Several systemic involvements have been reported in Covid-19 patients. In this study, it was aimed to investigate the cerebrovascular hemodynamics in patients with Covid-19.

**Materials and methods:**

The sample of this study included 20 patients hospitalized in our clinic diagnosed with Covid-19 via PCR modality and 20 healthy volunteers of similar age and sex. Bilateral middle cerebral arteries were investigated with transcranial Doppler ultrasonography. Basal cerebral blood flow velocities and vasomotor reactivity rates were determined and statistically compared.

**Results:**

When patient and control groups were compared, the mean blood flow velocity was found to be higher in Covid-19 patients than in the healthy volunteers and it was statistically significant (P = 0.00). The mean vasomotor reactivity rates values were found to be lower in the Covid-19 group than the healthy group and was also statistically significant (P = 0.00).

**Conclusion:**

An increase in basal cerebral blood velocity and a decrease in vasomotor reactivity rates in patients with Covid-19 can be considered as an indicator of dysfunction of cerebral hemodynamics in the central nervous system and this can be evaluated as a result of endothelial dysfunction.

## 1. Introduction

It came to attention that the number of pneumonia cases increased in December 2019 in Wuhan, China. As a result of the studies conducted a new virus belonging to the coronavirus family has been isolated as the cause of this disease, which has a high mortality rate despite influenza-like symptoms. Firstly, this virus was named 2019-nCoV, but in the following days it was identified as SARS-CoV-2. The disease that developed from this virus was called COVID-19WHO (2020). WHO Director-General’s remarks at the media briefing on 2019-nCoV on 11 February 2020 [online]. Website https://www.who.int/dg/speeches/detail/who-director-general-s-remarks-at-the-media-briefing-on-2019-ncov-on-11-february-2020 [accessed 28 June 2020]..

Coronaviruses (CoVs) such as influenza, are commonly seen in society. People affected with coronaviruses may be asymptomatic or have symptoms ranging from mild influenza-like symptoms to severe respiratory distress. Other viral outbreaks, such as Middle East Respiratory Syndrome (MERS) and Severe Acute Respiratory Syndrome (SARS) which both belong to the CoV family have recently occurred with varying clinical status [1–4]. 

Covid-19 spread rapidly all over the world and was declared as a pandemic by the World Health Organization (WHO) in March 2020. Advanced age, immunosuppression, or concomitant systemic diseases (such as hypertension, diabetes mellitus, cardiovascular diseases, malignancy etc.) were determined as the most important factors on mortality [5,6]. Symptoms occur approximately 5 days after viral exposure (incubation period) [7]. Fever, cough, and fatigue are seen in many patients while neurological symptoms such as headache, epileptic seizure, stroke, odor-taste disturbance, neuralgias, and changes in consciousness have also been reported [2,8,9]. Causes of neurological manifestations are not still fully understood.

Besides the respiratory symptoms, many systemic (neurologic, dermatologic, and gastrointestinal) manifestations may be seen in humans and these symptoms are sometimes the first and unique clues leading to the diagnosis of Covid-19s [10–13]. Endothelial dysfunction may be determined as the reason of these manifestations.

Transcranial Doppler (TCD) is a noninvasive ultrasonography modality that allows dynamic monitoring of cerebral blood flow velocity and its changes [14–16]. With this modality, cerebral blood flow cannot be directly demonstrated, but its velocity can be monitored. In this study, the aim was to investigate the cerebrovascular hemodynamics in patients with Covid-19 via TCD and to evaluate the vasomotor reactivity (VMR) capacity with the breath-holding index (BHI).

## 2. Materials and methods

The sample of this study included 20 patients hospitalized in our clinic diagnosed with Covid-19 via PCR modality and 20 healthy volunteers of similar age and sex. Patients needing intubation, who had history of performed cranial surgery, who had unsuitable temporal window for TCD, and pregnant patients were excluded from the study. Demographic data such as age and sex were recorded. TCD measurements of patients were evaluated immediately after hospitalization. All participants were informed about the examination and their written consent was obtained before the study. 

**Figure 1:**
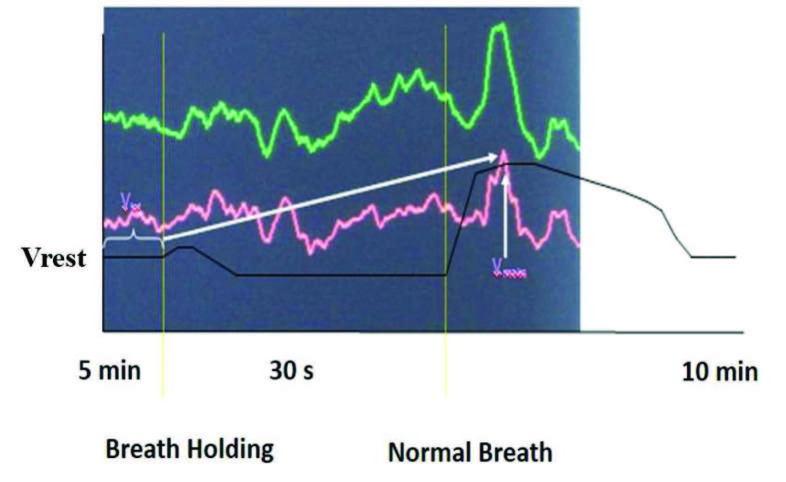
Cerebral blood flow changes during breath holding (Vrest: mean blood flow velocity in rest, Vmax: maximum blood flow velocity). Blood flow velocity is measured using TCD from the right (green) and left (red) middle cerebral arteries bilaterally.

**Figure 2:**
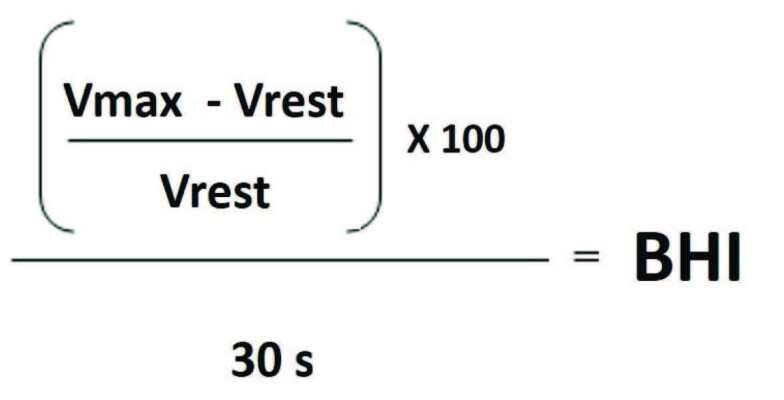
Calculation of breath-holding index (Vrest: mean blood flow velocity in rest, Vmax: maximum blood flow velocity).

DWL Multi-Dop T tool and QL software 2.8 were used for TCD examination. The middle cerebral arteries were insonated at an average depth of 45–60 mm through temporal bone window using 2 Mhz probes. After the vascular structures were determined bilaterally, two probes were fixed to the head with a frame. Patients were at rest for 10 min in a quiet room before the measurements were obtained. In order to evaluate mean basal cerebral blood velocity a 5-min record was obtained. The mean of blood flow velocity in these 5-min records was calculated and the average of these recordings was determined as the basal blood flow velocity. Afterwards patients were informed, and they were requested to hold their breath for 30 s to calculate the BHI. In the BHI study, the maximum point of blood flow velocity increases with the first breath taken after 30 s of breath-holding, and this was determined as the maximum speed. Cerebral blood flow velocity and BHI were calculated as shown in Figures 1 and 2.

For statistical analysis, SPSS 26.0 was used. Descriptive statistics were used for demographic data. Qualitative variables were indicated with frequency, and percentage and numerical variables were summarized by mean ± standard deviation. The Shapiro–Wilk test was used for determining normality. The Shapiro–Wilk Test is more appropriate for small sample sizes. For this reason, the Shapiro–Wilk test was used as a numerical means of assessing normality. In comparisons between groups Chi-square and Mann–Whitney U tests were used, and P < 0.05 is accepted as statistically significant.

## 3. Results

The study was conducted on 20 patients (13 male + 7 female) with diagnosed Covid-19 and 20 healthy volunteers (14 male + 6 female). The lowest age was 21 and the highest age was 81 in the patient group. The mean age of the patient group was 50.15 ± 19.07. In the control group, the lowest age was 21 and the highest age was 79. The mean age of the patient group was 47.90 ± 18.80. There was no significant difference between the two groups with respect to age and sex (P > 0.05). 

In the right and left middle cerebral arteries (MCAs), there were no significant differences between the patient and control groups in terms of both basal blood flow velocity values and VMR (P = 0.61, P = 0.89 vs. P = 0.24, P = 0.40, respectively) (Table). The mean blood flow velocity was found to be higher in the patient group than the control group and it was statistically significant (P = 0.01). The mean VMR values were found to be lower in the patient group than the control group and it was statistically significant (P = 0.01).

**Table:** Comparison between right and left MCAs in the patient and control groups in terms of BFL and VMR.

Covid-19 (mean ± sd)		P value	Healthy (mean ± sd)		P value
LMCA BFL	RMCA BFL		LMCA BFL	RMCA BFL	
61.32 ± 3.58	60.77 ± 4.82	0.61	56.94 ± 4.73	55.89 ± 5.45	0.24
LMCA VMR	RMCA VMR		LMCA VMR	RMCA VMR	
1.18 ± 0.03	1.17 ± 0.02	0.89	1.38 ± 0.01	1.36 ± 0.01	0.40

LMCA BFL: left middle cerebral artery blood flow velocity, RMCA BFL: right middle cerebral artery blood flow velocity, LMCA VMR: left middle cerebral artery vasomotor reactivity, RMCA VMR: right middle cerebral artery vasomotor reactivity.

## 4. Discussion

In this study, VMR evaluated with basal blood flow and BHI obtained from bilateral MCAs via TCD in patients with Covid-19 and healthy volunteers was investigated. According to the results, basal blood flow velocity rates of patients with Covid-19 were found to be higher than the healthy group. In contrast to the basal blood flow velocity, VMR values of patients with Covid-19 were found to be lower than the healthy group.

Cerebral autoregulation is a homeostatic process that maintains cerebral blood flow at regular intervals, despite fluctuations in cerebral perfusion pressure [17]. Changes in the vascular tonus have a key role in ensuring cerebral hemodynamics. Cerebral blood flow is particularly sensitive to blood carbon dioxide exchange, but in some cases where the level of carbon dioxide remains constant, it is known that cerebral blood flow can be regulated by adjusting the heart rate or peripheral circulation [18]. 

The mechanisms of cerebral autoregulation remain poorly understood, especially in humans. Generally, three different mechanisms, metabolic, myogenic, and neurogenic are thought to contribute to the process of cerebral autoregulation. These mechanisms affect the cerebral blood flow and this provides the regulation [19]. VMR provides information about the capacity of the cerebral autoregulation and can be evaluated with several modalities, such as the BHI method.

TCD is a noninvasive, reproducible, bedside practicable diagnostic tool that can demonstrate the blood flow velocity and direction in major intracranial arteries. Cerebral arterial autoregulation is constituted with changes in the diameters of small arteries. There are no significant changes in the diameters of these vessels during normal pressure changes, or the constituted changes are negligible. Therefore, relative blood flow alterations resulting from diameter changes in small vessels can be evaluated as an autoregulation response [19,20]. 

Several studies have been made regarding cerebral autoregulation, in particular in patients with migraine, and changes in VMR and basal blood flow velocity were observed in many of these studies [21,22]. In the Rotterdam study, which was conducted by Portegies et al., decreased VMR results were found to be related to mortality. Authors reported that stroke was independent of this relationship and the decreased VMR values were based on impairment of the vascular system [23]. Similarly to this study, Ju et al., also reported that decreased VMR is an important prognostic factor for stroke [24]. In one study carried out by Mamontov et al., the low neurogenic reactivity against increased peripheral vascular tonus was found to be the worst prognostic factor in hypertensive patients [25]. 

Hypertension is one of the most important factors of poor prognosis in patients with Covid-19 [5,6]. In addition, many different studies have shown that patients with Covid-19 tend to thrombosis, and also stroke cases have been reported in these studies [26–32]. In particular in cases related to thrombus, an underlying endothelial dysfunction has been accused. Varga et al. have histopathologically shown that the SARS-CoV-2 virus caused endothelial damage [33]. In addition to endothelial physical barrier function, it also has paracrine, endocrine, and autocrine implications. In this way, it affects the vascular tonus and provides the vascular homeostasis [34]. Endothelial damage disrupts the balance in the vascular tonus and it causes the ischemia, edema, and procoagulant state in the organ in which it is located [35]. 

As a result of this study, an increase in cerebral basal blood velocity and a decrease in VMR was found in patients with Covid-19. It is thought that this can be evaluated as a result of endothelial dysfunction in the vascular structures of central nervous system.

## Informed consent

This study was approved by the Ministry of Health (Protocol No: 2020-05-04T10-38-53) and the Gülhane Training and Research Hospital Ethic Committee (Protocol No: 2020-07-165).
